# Supplementary material for: Orbital-flop Induced Magnetoresistance Anisotropy in Rare Earth Monopnictide CeSb
Source: Nat Commun. 2019 Jun 28;10:2875. doi: 10.1038/s41467-019-10624-z (PMC6599061; doi:10.1038/s41467-019-10624-z)
Supplement: Supplementary file 1 — Supplementary Information [file 41467_2019_10624_MOESM1_ESM.pdf]

Supplementary Information

Orbital-flop Induced Magnetoresistance Anisotropy in Rare Earth Monopnictide CeSb

by Jing Xu et al.

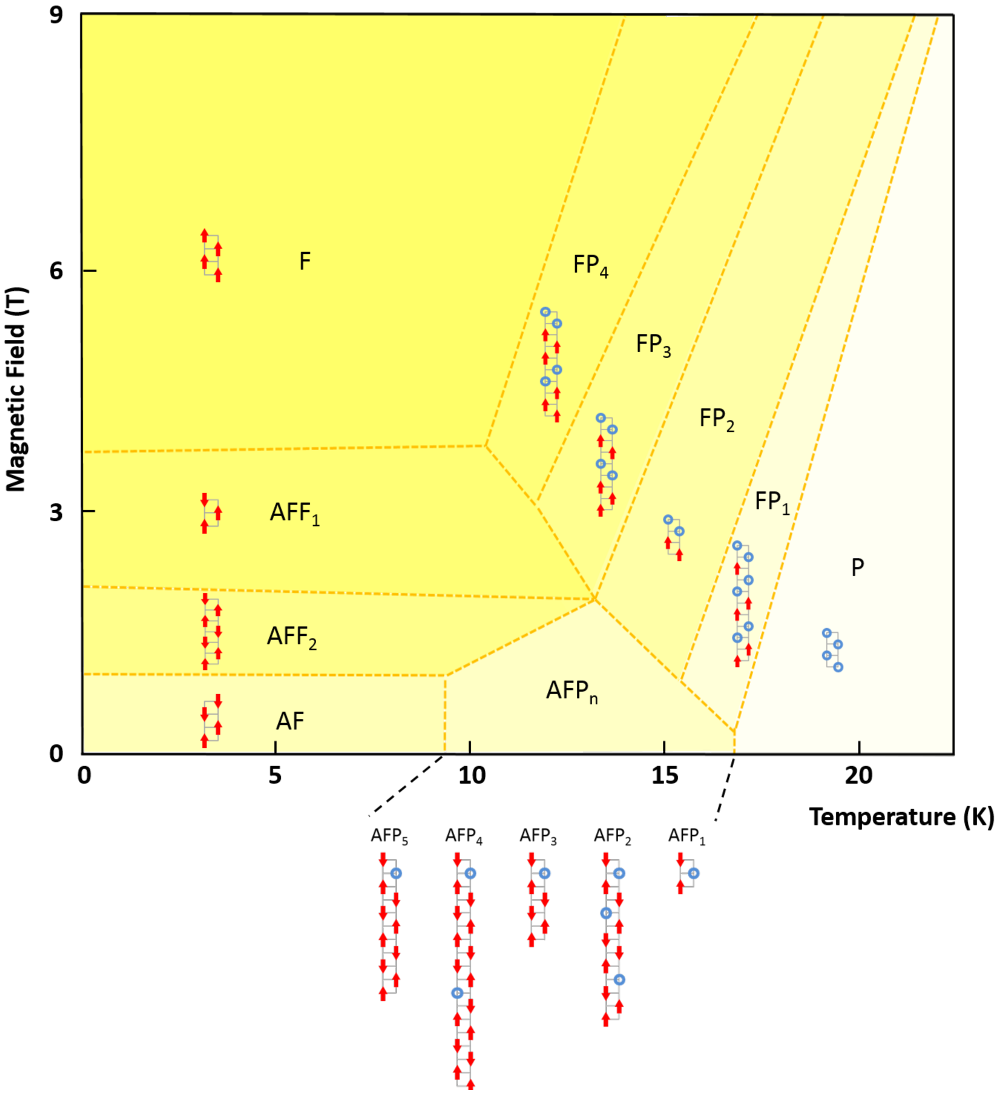

Supplementary Fig.1 | Magnetic phase diagram of CeSb. There are more than 14 phases indicated by the schematic representations of magnetic structure for the Ce layers, where red arrows indicate ordered Ce-ion layers with magnetic moments of $2\mu_B$/Ce and blue circles indicate paramagnetic ones. These states are made up of stacks of magnetic planes. Each state has a particular stacking order of ferro- and antiferromagnetically aligned layers intermixed with paramagnetic layers [1,2]. Potential new phases were also identified between F and AFF1 in Ref.1 and between AFF2 and AFF1 in Ref.1 and Ref.2. The magnetic field is applied along the [001] direction.

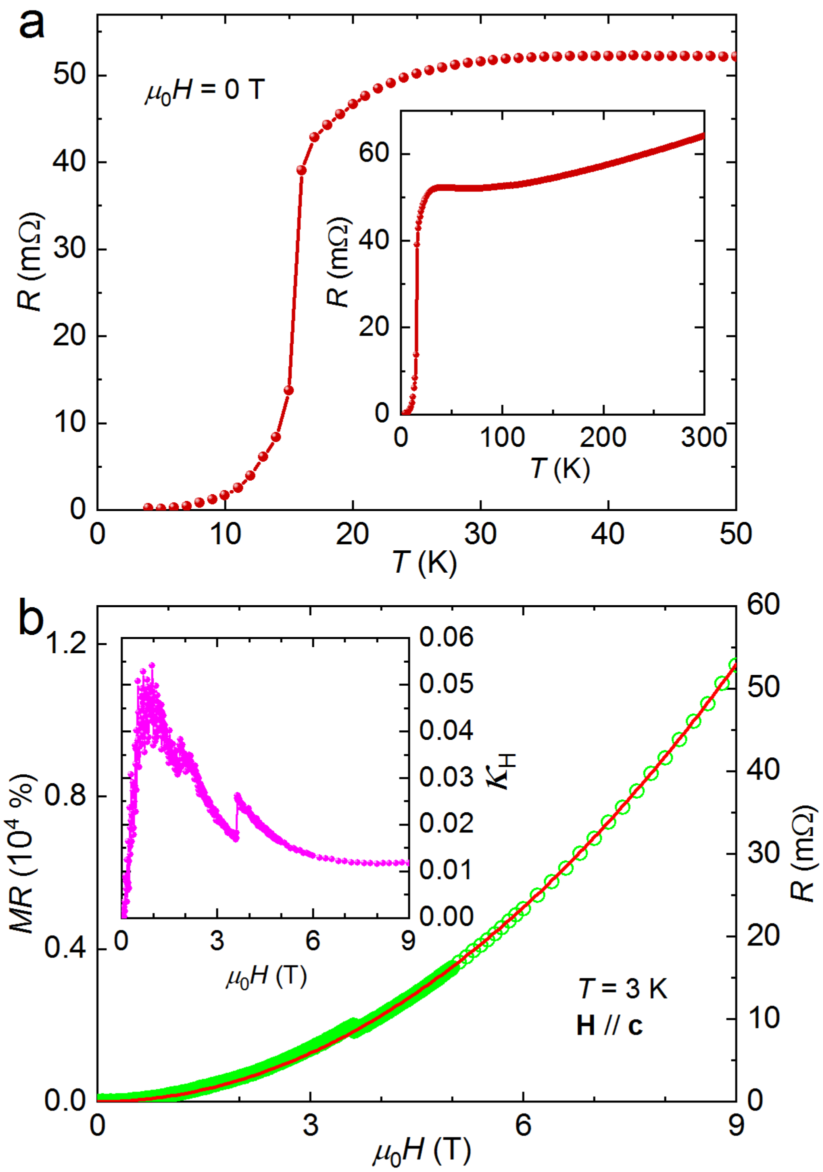

Supplementary Fig.2 | Basic characterization of the CeSb crystal. **a**, temperature dependence of the resistance in the absence of a magnetic field, indicating a magnetic ordering temperature T_N ≈16 K. The inset show data up to room temperature, demonstrating a residual resistance ratio *rrr* ≈ 140. **b**, Magnetic field dependence of the resistance at 3 K and **H || c**, revealing extremely large magnetoresistances with $MR \approx 1.1\times10^4$ % at 9 T, where *MR* is defined as $MR = [R(B) - R_0]/R_0$ with R_0 being the sample resistance in the absence of an external magnetic field. The apparent kink at ~ 3.62 T comes from an antiferromagnetic to ferromagnetic transition. The inset of (b) presents the magnetic field dependence of the Hall factor $\kappa_H = (\rho_{xy}/\rho_{xx})^2$. Since $\kappa_H \ll 1$, the magnetoresistance should follows $MR = (\mu_{avg}H)^n$ with $n \approx 2$, where μ_{avg} is the average mobility of the charge carriers [Ref.3], as indicated by the red curve in **b** where μ_{avg} =1.19 $m^2V^{-1}s^{-1}$ and n = 2 are used. The data were obtained from Sample A.

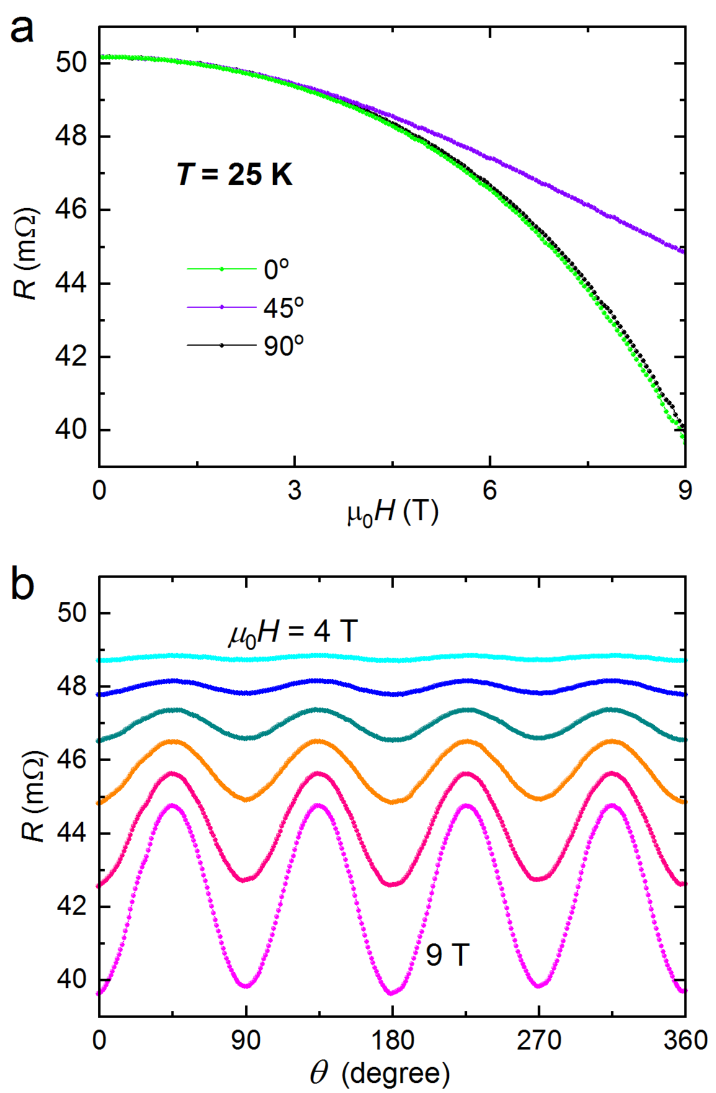

Supplementary Fig.3 | Anisotropic magnetoresistance in the paramagnetic state. **a**, Magnetic field dependence of the resistances at 25 K and θ = 0° (**H**∥**b**), 45° and 90° (**H**∥**c**). **b**, Angle dependence of the resistance at 25 K and $\mu_0 H$ = 9 T to 4 T at intervals of 1 T, showing a four-fold symmetry expected from the anisotropic Fermi surface. The data were obtained from Sample A.

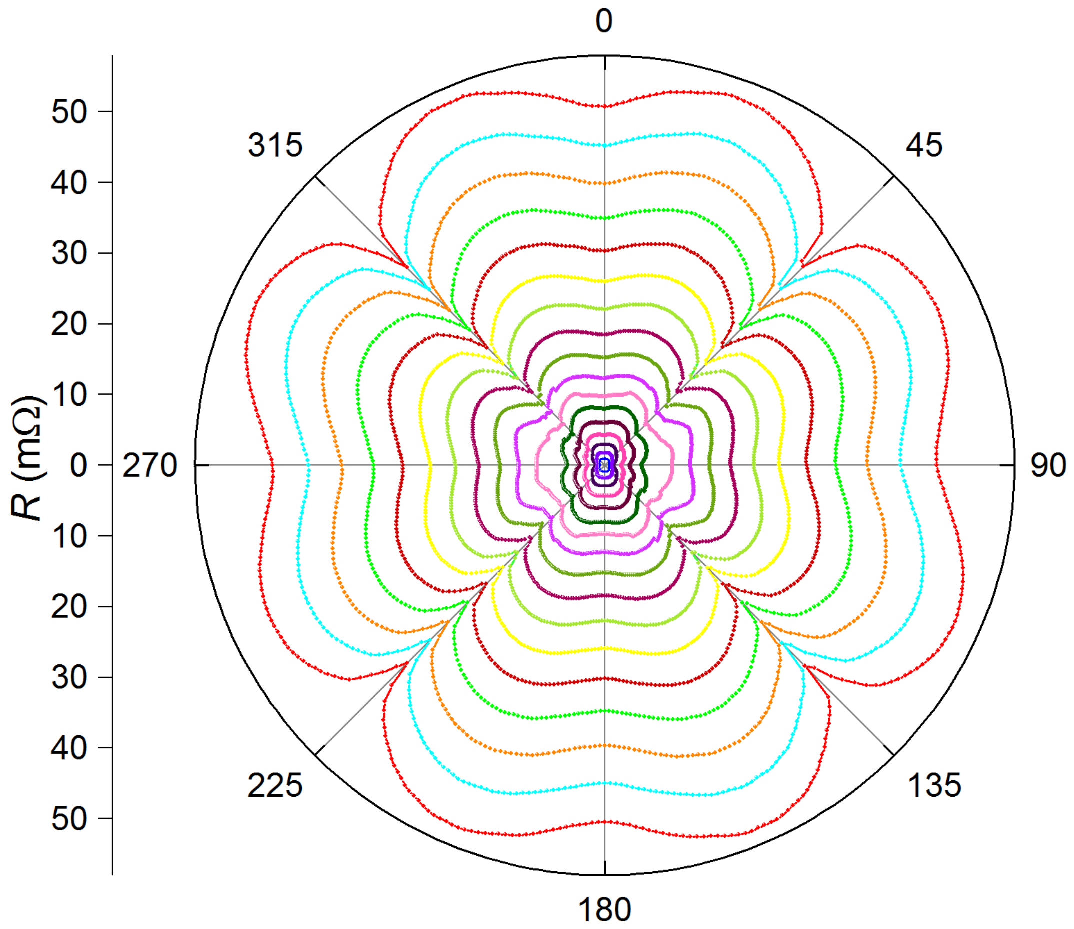

Supplementary Fig.4 | Angle dependence of the magnetoresistance. Data obtained from Sample A at $T = 5$ K at $\mu_0 H = 9$ T to 1 T in intervals of 0.5 T. The magnetoresistances show the same angle and magnetic field dependences as those obtained in Fig.1b and Fig.1c for $T = 3$ K. Minima can be clearly identified at $\theta = 45°$, 135°, 225°, and 315° in the curves for $\mu_0 H \geq 5$ T, confirming the finding presented in Fig.1.

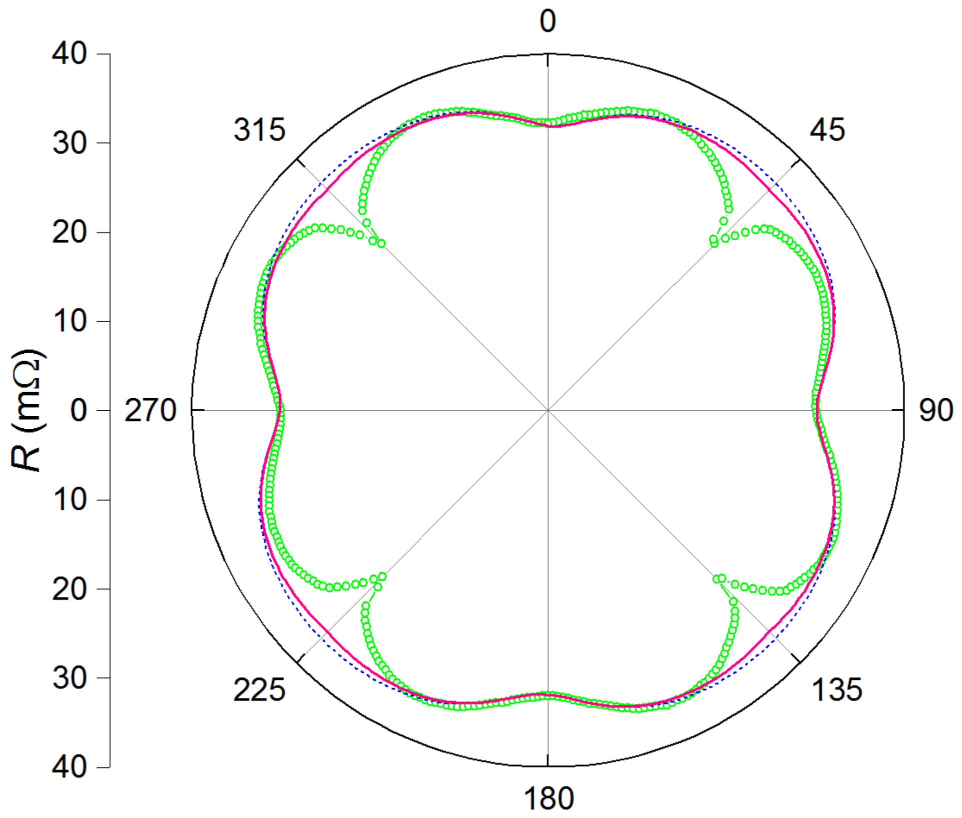

Supplementary Fig.5 | Comparison of the experimental and calculated angle dependence of the magneto-resistance. Symbols are data taken from Sample A at $T = 3$ K and $\mu_0 H = 7$ T. The dashed blue and solid red curves represent the calculated results for isotropic $B = \mu_0 H + \mu_0 M_0$, i.e. for **M** always parallel to **H**, and anisotropic $B = \mu_0 H + \mu_0 M_0 \cos\varphi$, i.e., for the orbital-flop case, respectively. $\mu_0 M_0 = 0.38$ T was used for a ferromagnetic CeSb with a magnetization of 2 μ_B/atom, where μ_B is Bohr magneton. Following the analysis procedures in Ref.4, we used two elliptical electron pockets with an anisotropy of $\lambda_\mu = 4$ and one isotropic hole pocket. The derived mobilities are $\mu_{//} = 0.4$ and 0.6 $m^2V^{-1}s^{-1}$, $\mu_\perp = 6.4$ and 9.6 $m^2V^{-1}s^{-1}$ for electrons, and $\mu_h = 0.85$ $m^2V^{-1}s^{-1}$ for holes. These values are very close to those of LaSb [Ref.4]. The different electron mobilities in the two perpendicular pockets are required to account for the asymmetry in the experimental $R(\theta)$ curve, which could originate from the lattice distortion as discussed in the text. To calculate the red curve we replace $B_\theta = \mu_0 H + \mu_0 M_0$ in the formula used to derive the dashed blue curve with $B_\theta = [(\mu_0 H)^2 + (M_0)^2 + 2\mu_0 H M_0 cos\varphi]^{1/2}$, while keeping other parameters unchanged (see text for the relationship between φ and θ). Clearly, the magnetization anisotropy induced by the orbital-flops does not account for the pronounced suppression of the magnetoresistances at angles close to $\theta = 45°$, 135°, 225° and 315°. We propose that multi-domain states exist in the orbital-flop region, resulting in further reduction of the magnetoresistance. This is because the of electron mobility μ decreases due to the scattering of the domain walls and the magnetoresistance MR depends on the mobility μ, with a typical relationship of $MR \sim \mu^2$ (see Supplementary Figure 2).

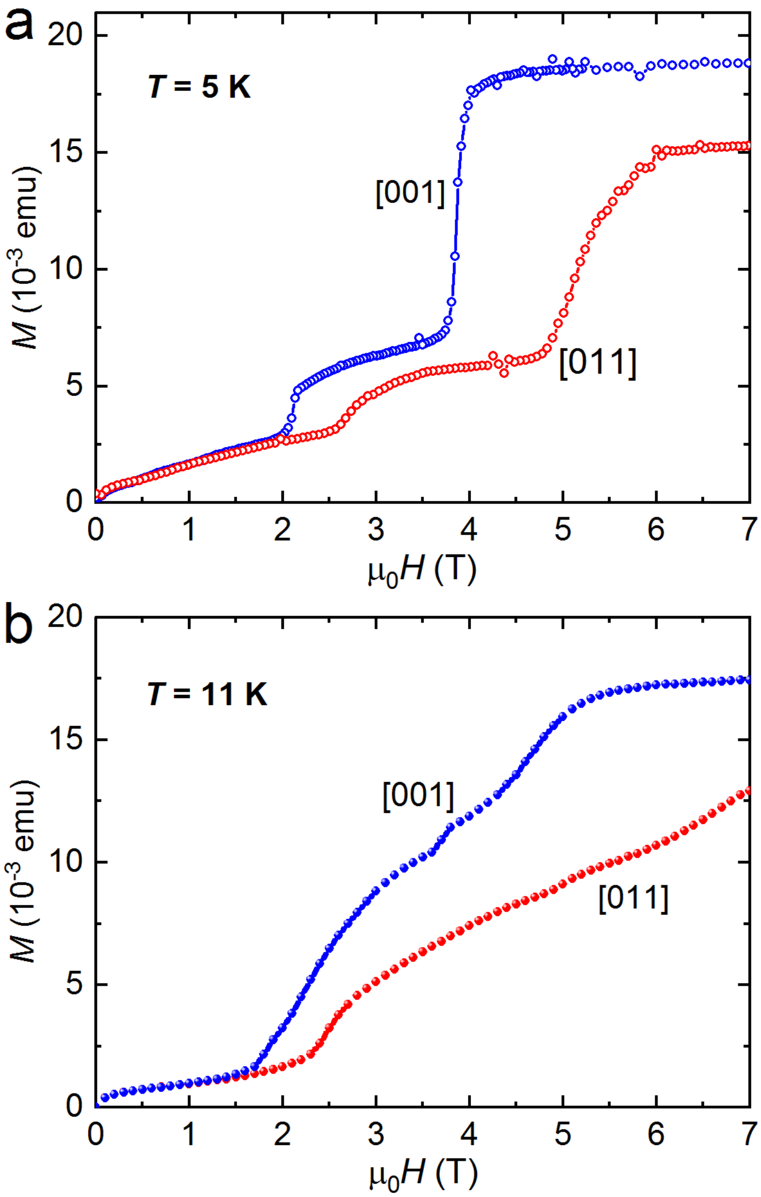

Supplementary Fig.6 | Magnetic field dependence of the magnetization at two magnetic field orientations. **a**, Results for $T = 5$ K. **b**, Curves for $T = 11$ K. The data were taken from Sample C with decreasing magnetic field.

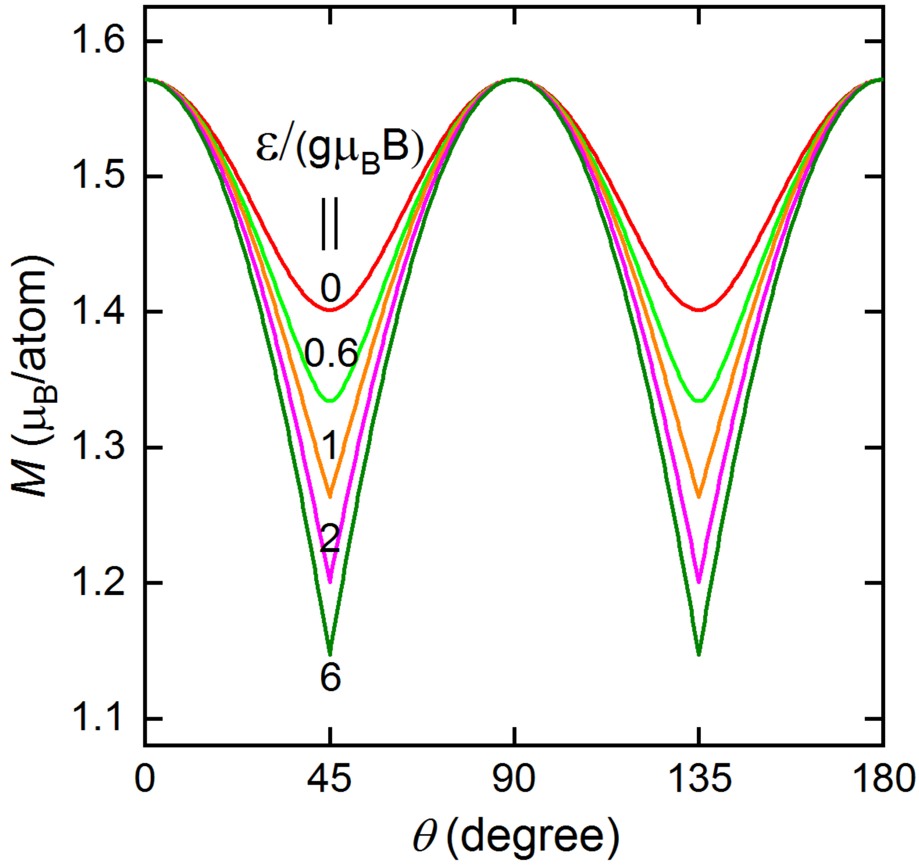

Supplementary Fig.7 | Effect of the strength of the ferromagnetic coupling on the magnetization anisotropy. Angle dependence of the magnetization at various ferromagnetic coupling constant $\varepsilon/(g\mu_B B)$.

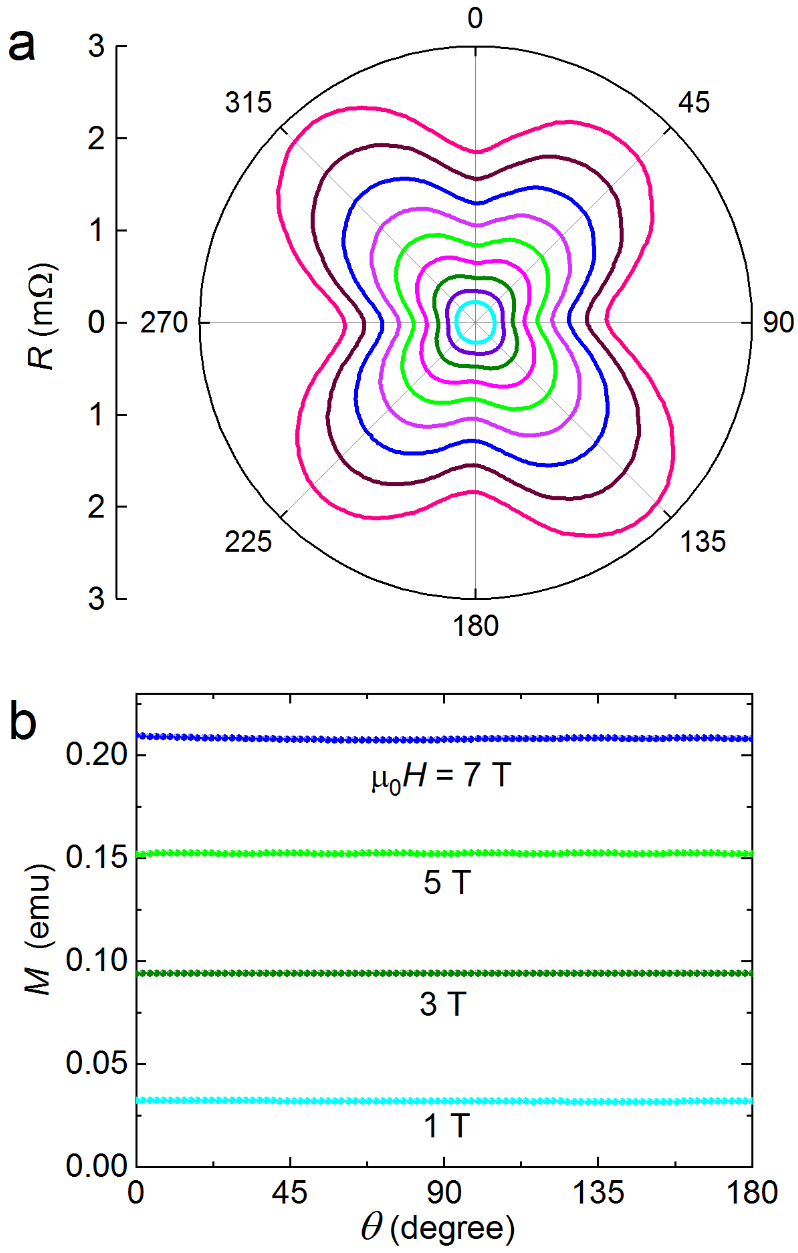

Supplementary Fig.8 | Anisotropy of the magnetoresistance and magnetization of a GdBi crystal. **a**, Angle dependence of the resistance at 3 K and $\mu_0 H$ = 9 T to 1 T at intervals of 1 T, showing four-fold symmetry expected from the anisotropic Fermi surface. **b**, Angle dependence of the magnetization at 5 K and $\mu_0 H$ = 7 T to 1 T at intervals of 2 T. GdBi is an orbitally quenched system and hence has different angle dependences of the magnetoresistance and magnetization from those (Fig.1 and Fig.4) of CeSb with orbital-flops.

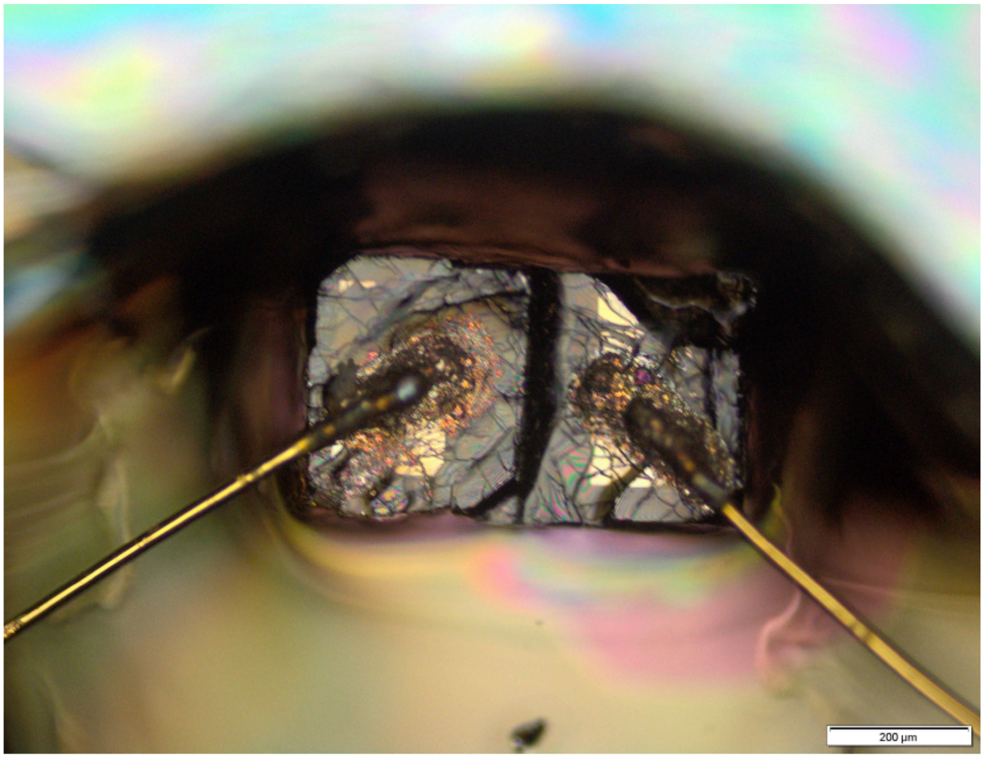

Supplementary Fig.9 | Optic image of Sample B with two contacts. As indicated by the schematic in Fig.4a, a 30 nm thick Py layer with a gap in the middle is sputter-deposited onto the surface of the CeSb crystal to form two Py/CeSb spin-valve-like structures. A 20 nm thick layer of Au was coated onto the Py layer to improve the adhesion of silver paste that is used to attach two gold wires of 50 µm for electrical connections to the current source and the nanovoltmeter. (The image was taken after the experiments. Damage to the contacts occurred when removing the sample from the holder.)

Supplementary References

1. Ye, L., Suzuki, T., Wicker, C. R. &. Checkelsky, J. G. Extreme magnetoresistance in magnetic rare-earth monopnictides. *Phys. Rev. B* **97**, 081108(R) (2018).
2. Wiener, T.A. & Canfield, P.C. Magnetic phase diagram of flux-grown single crystals of CeSb. *J. Alloys Compd.* **303–304**, 505–508 (2000).
3. Xu, J. et al. Reentrant metallic behavior in the Weyl semimetal NbP. *Phys. Rev. B* **96**, 115152 (2017).
4. Han, F. et al. Separation of electron and hole dynamics in the semimetal LaSb. *Phys. Rev. B* **96**, 125112 (2017).
